# High Throughput Identification of Monoclonal Antibodies to Membrane Bound and Secreted Proteins Using Yeast and Phage Display

**DOI:** 10.1371/journal.pone.0111339

**Published:** 2014-10-29

**Authors:** Lequn Zhao, Liang Qu, Jing Zhou, Zhengda Sun, Hao Zou, Yunn-Yi Chen, James D. Marks, Yu Zhou

**Affiliations:** 1 Department of Anesthesia and Perioperative Care, University of California San Francisco, San Francisco General Hospital, San Francisco, California, United States of America; 2 Departments of Pathology & Laboratory Medicine, University of California San Francisco, San Francisco, California, United States of America; CIP, NCI-Frederick, NIH, United States of America

## Abstract

Antibodies are ubiquitous and essential reagents for biomedical research. Uses of antibodies include quantifying proteins, identifying the temporal and spatial pattern of expression in cells and tissue, and determining how proteins function under normal or pathological conditions. Specific antibodies are only available for a small portion of the proteome, limiting study of those proteins for which antibodies do not exist. The technologies to generate target-specific antibodies need to be improved to obtain high quality antibodies to the proteome at reasonable cost. Here we show that renewable, validated, and standardized monoclonal antibodies can be generated at high throughput, without the need for antigen production or animal immunizations. In this study, 60 protein domains from 24 selected secreted proteins were expressed on the surface of yeast and used for selection of phage antibodies, over 400 monoclonal antibodies were identified within 3 weeks. A subset of these antibodies was validated for binding to cancer cells that overexpress the target protein by flow cytometry or immunohistochemistry. This approach will be applicable to many of the membrane-bound and the secreted proteins, 20–40% of the proteome, accelerating the timeline for Ab generation while reducing the cost.

## Introduction

Availability of antibodies (Abs) strongly determines which proteins of the proteome are studied [1]. Over half the human proteome is not annotated, and functional Abs are not reliably available for these proteins. Even when monoclonal or polyclonal Abs are commercially available, a high proportion of these Abs show either poor specificity or fail to recognize their targets [2]–[6]. For example, a recent editorial by Michel et al. highlighted the lack of target specificity for 49 Abs against 19 subtypes of GPCRs [7]. An additional problem is lot-to-lot variability in Ab specificity, including monoclonal Abs (mAbs) made via hybridoma technology, resulting in inconsistent assay results [5].

Among the proteome, the secretome includes membrane-bound and extracellular proteins that are processed through the secretory pathway [8]. Secreted proteins are involved in a myriad of normal functions [8]–[11], as well as in disease processes [12], [13]. This class of proteins is extensively studied for their roles in the pathogenesis of disease, as diagnostic and prognostic biomarkers and as targets of therapeutics [12], [14]. As of May 2014, 39 of the 40 FDA approved Abs target proteins in a subset of the human secretome [15]–[17]. This is also true of the majority of the more than 338 therapeutic Abs under clinical development. Secreted proteins are ideal candidates for a high throughput recombinant Ab (rAb) generation platform because they are frequently implicated in disease pathogenesis, and because expression and purification of these types of proteins for use in Ab generation is challenging. Secreted proteins generally do not fold properly in the bacterial cytosol, necessitating use of the bacterial secretion system for expression. The presence of multiple disulfide bonds in the extracellular proteins and in the extracellular domains of type 1 and type 2 membrane proteins is typical, and their large size makes expression yields in bacteria frequently too low to be useful [18]. This can be partially overcome by expressing isolated protein domains. Although expression in either insect or mammalian cells is often required but these are difficult systems to automate and expression yields are variable [19]. Multi-pass transmembrane proteins are even more difficult to express and purify. Because of the large hydrophobic transmembrane domains, they must be harvested from membrane fractions and purified in the presence of detergents [20], [21]. It is not uncommon for them to denature during purification making recognition of the native conformation unlikely. Furthermore, many of these proteins are evolutionarily conserved, limiting the robustness of the immune response when the protein is used as an immunogen [22].

Yeast display is an attractive platform for generating antigens for phage Ab selections as no antigen purification is required [23]. Yeast display is a robust system for displaying a variety of different proteins in their properly folded states on the yeast surface. The antigen of interest is fused to either the N- or C-terminus of the yeast Aga2 protein which disulfide bonds to the Aga1 membrane protein. A flexible linker between the antigen of interest and Aga2 ensures accessibility of the antigen to the Abs. Domains of human EGFR, T-cell receptor, NY-Eso-1, breast cancer antigens, and botulinum neurotoxin have been functionally displayed on the yeast surface and used to map Ab epitopes [24]–[27] [28].

Here we report a high throughput scalable approach to generate widely available, renewable, validated and standardized sets of recombinant Ab reagents (rAbs) to plasma membrane and extracellular proteins. We demonstrate the generality of our approach by generating Abs to a variety of protein classes. A key benefit of the technology is that the expensive, time-consuming and tedious task of antigen generation and purification is bypassed by displaying the antigen at high levels on the surface of yeast. Antigens expressed on yeast were used for selection of phage Abs as well as for validation and characterization. The use of phage display bypasses the low throughput, time-consuming, and expensive immunization of animals to generate polyclonal Abs or the use of hybridoma technology to generate mAbs. Moreover, the Ab genes are cloned, and the rAbs are forever renewable and can easily be formatted for expression as Ab fragments or traditional mAbs with any Fc.

## Results

### Display of membrane and secreted proteins on the surface of yeast cells

**Figure 1:**
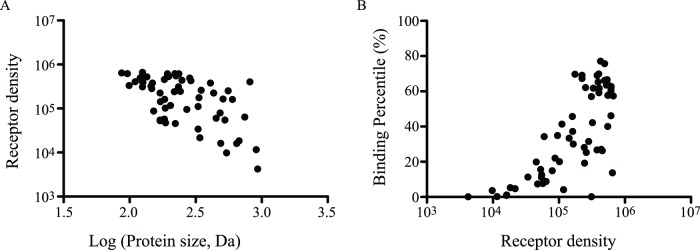
Yeast surface display of proteins and results of phage antibody library selections. A) The density of each protein domain on the induced yeast surface was quantitated and plotted against the domain size; B) The percentage of the polyclonal phage output binding the target antigen is shown as a function of antigen receptor density on the yeast surface.

Sixty different cDNA from 17 single pass, 1 multi-pass transmembrane, 2 GPI-anchored, and 4 secreted proteins were displayed on the surface of yeast (Table 1). These included 14 different single domain classes, multiple domains, and full-length extracellular domains (ECD). Domains were identified based on experimentally determined or homology-modeled structures, and cloned into the yeast display vector pYD2. Display was induced and quantitated using the SV5 tag at the C-terminus of the displayed protein with 86% of the proteins showing detectable display above the background (Fig. 1a). The display level was weakly inversely correlated with the protein size (Fig. 1a). Some protein domains were poorly expressed, such as the semaphorin domain of c-Met.

**Table 1:** Secretome proteins used for yeast display and phage antibody selection.

Gene Name	Display	Domain Classes	Yeast Display Strategy(# residues displayed)
EGFR	Type 1 TM	Receptor L domain, Furin-likecysteine rich region, Furin-like repeats	4 Individual domains (185, 125, 171, 135)
HER2	Type 1 TM	Receptor L domain, Furin-likecysteine rich region, Furin-like repeats	4 Individual domains (193, 126, 170, 145)
HER3	Type 1 TM	Receptor L domain, Furin-likecysteine rich region, Furin-like repeats	4 Individual domains (187, 124, 170, 147)
HER4	Type 1 TM	Receptor L domain, Furin-likecysteine rich region, Furin-like repeats	4 Individual domains (183, 125, 170, 152)
EPHA2	Type 1 TM	Ligand Binding Domain, SAM(Sterile alpha motif), FN3 domain	Full-length ECD (510)
EPHB3	Type 1 TM	Ligand Binding Domain, SAM,FN3	Full-length ECD (527)
VEGFR2	Type 3 TM	Ig	ECD (744) & Ig2-3 (204)
FGFR1	Type 1 TM	Ig	ECD (348), Ig1 (87), Ig2-3 (218)
c-Met	Type 1 TM	Sema (semaphorin domain), PSI(Plexin repeat), IPT (Ig-like,plexins, transcription factors)	Full-length ECD & individual domain combinations (489, 908, 271, 340)
MST1R	Type 1 TM	Sema (semaphorin domain), PSI, IPT	ECD & 3 individual domain combinations (933, 647, 222, 330)
ICAM1	Type 1 TM	Ig	Ig1-5 (452) & Ig1 (99)
PECAM	Type 1 TM	Ig	Ig1-2 (236)
VCAM	Type 1 TM	Ig	Ig2-7 (673) & Ig2-3 (197)
EpCAM	Type 1 TM	Thyroglobulin type-1	Full-length ECD (242)
E-Cad	Type 1 TM	Cadherin like domain	Cad domains 1-5 (542), 1-2 (220)
CD44	Type 1 TM	Link domain	7 variant domains (149, 409, 558, 249, 291, 335, 433)
CD47	5 TM	Ig-like V-type	N-terminal ECD 1 (123)
CD73	GPI anchor	N-terminalmetallophosphatasedomain	Full-length ECD (523)
CD168	GPI anchor	Hyaluronan-bindingfragment	63 kDa isoform (561)
MSLN	Secreted	No domain superfamily	Cleaved form (285)
MMP9	Secreted	Fibronectin type-II,Hemopexin	82 kDa (601) & FNII 1-3 (184)
TIMP1	Secreted	NTR	Full-length protein (183)
TIMP2	Secreted	NTR	Full-length protein (193) & NTR (125)
Robo1	Type 1 TM	Ig-like C2-type,Fibronectin type-III	Full-length ECD & individual domain combinations (96, 110, 330, 484, 814)

### Selection of phage single chain (scFv) antibody library on yeast displayed proteins

**Figure 2:**
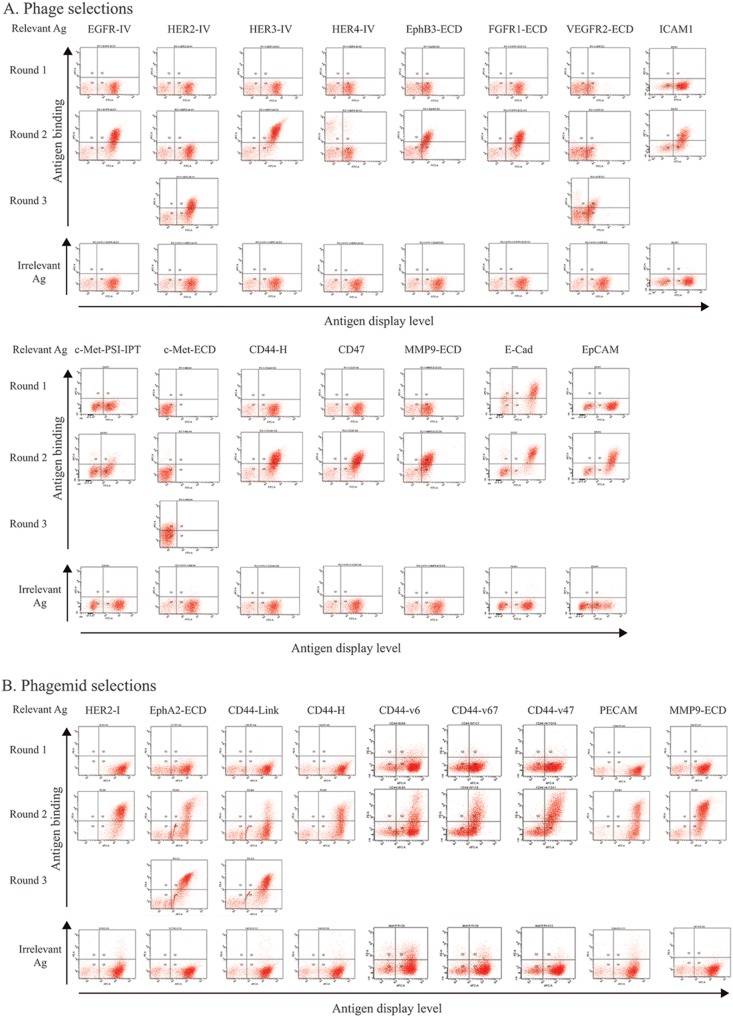
Selection enrichment demonstrated by flow cytometry analysis of polyclonal phage binding to target antigen expressing yeast cells. Binding of fifteen polyclonal phage Ab outputs (A) and nine phagemid Ab outputs (B) from the first, second and third round of selection compared to binding to an irrelevant yeast displayed antigen. The antigen used for selection is shown above each dot-plot.

To determine whether phage antibodies could be successfully selected on yeast-displayed antigens, a naïve human scFv multivalent phage display library [29], [30] was selected on each of the 60 yeast-displayed protein domains. After three rounds of selection, the polyclonal phage outputs were analyzed for binding to the target protein domains displayed on yeast cells by flow cytometry and a percentage of antigen binding phage above 10% used as an indicator of successful antibody selection. Of 60 selections, 49 specifically bound the yeast displayed target antigen compared to the un-induced yeast cells (Fig. 1b, Fig. 2a). On average, 47% of the polyclonal phage from the selections bound the yeast-displayed antigens, with a range of 11% to 77%. Of the failed selections, 9 were from proteins that were poorly displayed. Since most proteins were represented by more than one domain, there was at least one domain from each of the 24 target antigens that displayed and resulted in antigen binding phage antibodies. The exception was the antigen NRP1, which was only displayed as full-length ECD. The correlation between protein density on the yeast surface and the percentage of target binding phage suggested that display levels greater than 50,000 copies per yeast cell are required for successful phage selections. However, good display was insufficient by itself, since some of the better-displayed protein domains failed to generate Abs, such as FGFR1 Ig domain 1, NRP1, and VEGFR2 Ig domain 2&3 (Fig. 1b).

A naïve scFv phage display library in the monovalent format (scFv) was also selected on 9 of the yeast-displayed protein domains, including ErbB2 domain1, EphA2 ECD, PECAM domain 1-2, MMP9 FN domain 1-3, and 5 CD44 variants (link, H, v6, v67, and v47) (Fig. 2b). All nine selections yielded an antigen binding polyclonal phage population.

### Characterization of phage antibodies

From the multivalent phage library selections, the initial screening of 96 random clones from each of the 26 polyclonal phage outputs yielded 1152 mAbs that bound the yeast-displayed antigens. DNA sequencing revealed 162 unique sequences for Abs binding the 26 yeast-displayed domains (Table 2). Flow cytometry analysis and RCA amplification for DNA sequencing using 96-well microtiter plate allowed the screening to be completed in 3 weeks. From the monovalent phage library selections, the nine successful selections yielded 345 mAbs of which 108 were unique sequences (Table 2).

**Table 2:** Results of phage antibody selections.

Multivalent phagelibrary	Domain	Binding Abs(per 95 screened)	Unique mAbsidentified	Cell binding (cell line and number of mAbs)
EGFR	III	62	1	A431 (1)
	IV	54	2	A431 (2)
ErbB2	I	90	2	AU565 (2)
	II	15	1	AU565 (1)
	III	11	1	AU565 (1)
	IV	5	6	AU565 (1)
ErbbB3	IV	59	12	N.D.
ErbbB4	IV	42	10	N.D.
EphA2	ECD	50	3	MDAMB231 (3)
EphB3	ECD	53	10	MDAMB453 (5)
VEGFR2	ECD	10	4	N.D.
FGFR1	Ig2-3	78	4	N.D.
	ECD	57	9	N.D.
c-Met	PSI-IPTs	16	4	MDAMB231 (2)
NRP1	ECD	20	2	N.D.
ICAM1	Ig1	76	14	SUM159PT (5)
	Ig1-5	30	6	SUM159PT (3)
PECAM 1	Ig1-2	21	3	N.D.
EpCAM	ECD	67	13	N.D.
E-Cad	Cad1-2	2	2	N.D.
CD44	Link	60	6	MDAMB231 (1)
	H	76	2	N.D.
CD47	ECD1	60	16	N.D.
MMP9	FN1-3	51	16	N.D.
	ECD	22		N.D.
hFc	CH2-CH3	65	13	N.D.
Monovalent phage library				
ErbB2	I	63	8	SKBR3 (5)
EphA2	ECD	57	18	MDAMB231 (10)
CD44	Link	16	2	MDAMB231 (2)
	H	40	6	MDAMB231 (1)
	V6	11	5	JIMT (1)
	V67	34	13	JIMT (1)
	V47	48	29	JIMT (4)
MMP9	FN1-3	50	17	N.D.
PECAM 1	Ig1-2	26	10	N.D.

N.D. This test was not done.

Antigen and antigen domain used for selection are indicated along with the number of binding mAbs, the number of unique mAbs, and the number of mAbs binding mammalian cells expressing the target antigen. The specific cell line used is also indicated.

Phage antibody libraries in the monovalent and multivalent forms selected on six yeast-displayed domains were also compared with regard to the selection enrichment and the number of unique antibodies. Although the multivalent fd phage library presents multiple copies of scFv potentially favoring antigen binding compared to the monovalent phagemid library, no obvious differences in enrichment of binding phage antibodies was observed (Figure 2). However, more unique sequences were identified from the phagemid library selections than from the fd library selections (Table 2), perhaps reflecting its over 10-fold greater size compared to the multivalent fd phage library.

**Figure 3:**
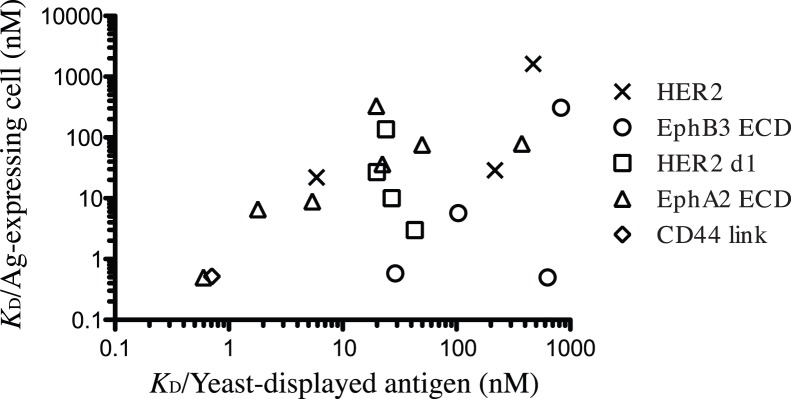
Binding affinities of scFv antibodies. The *K*_D_ of purified scFv antibody was measured on yeast displayed antigen domains and compared with the *K*_D_ for binding to antigen (Ag) expressing mammalian cells by flow cytometry. scFv mAbs were incubated with the yeast cells transformed and induced for antigen expression, or with the antigen overexpressing cancer cells; SKOV3 cells for HER2 mAbs, MDA-MB-231 for EphA2 and CD44 mAbs, Colo205 for EphB3 mAbs.

Of the 270 unique mAbs from 35 selections (26 from the multivalent fd phage and 9 from the monovalent phagemid library), over 100 antibodies were evaluated for binding to seven of the target antigens using intact mammalian cell lines known to overexpress the target antigen. This screening identified 49 mAbs recognizing the cell surface target antigen as expressed on the mammalian cell surface. The fraction of mAbs that stained the cells specifically varied (Table 2), suggesting that in some cases the protein domains were not displayed on the yeast cell surface in their native conformations. For 19 single chain Fv (scFv) mAbs, the binding affinities were evaluated by measuring the *K*_D_ on both the yeast-displayed antigens and the antigen overexpressing cells in order to show that 1) the affinities measured on yeast are comparable to affinities on antigen in its “native” state; and 2) that the antibody affinities are in the expected range. The affinity of binding to mammalian cell surface proteins was measured using flow cytometry for 19 purified scFv antibodies binding 4 different antigens (Her2, EphA2, EphB3 and CD44). Affinities generally correlated with those measured on the yeast displayed antigen and 12 of them were less than 30 nM (Fig. 3). The correlation coefficient for these two measurements was 0.47. Variations in some *K*_D_ values may reflect the conformational differences between the epitopes displayed on yeast surface and that on the cancer cells (Fig. 3).

### Validation of antibodies

**Figure 4:**
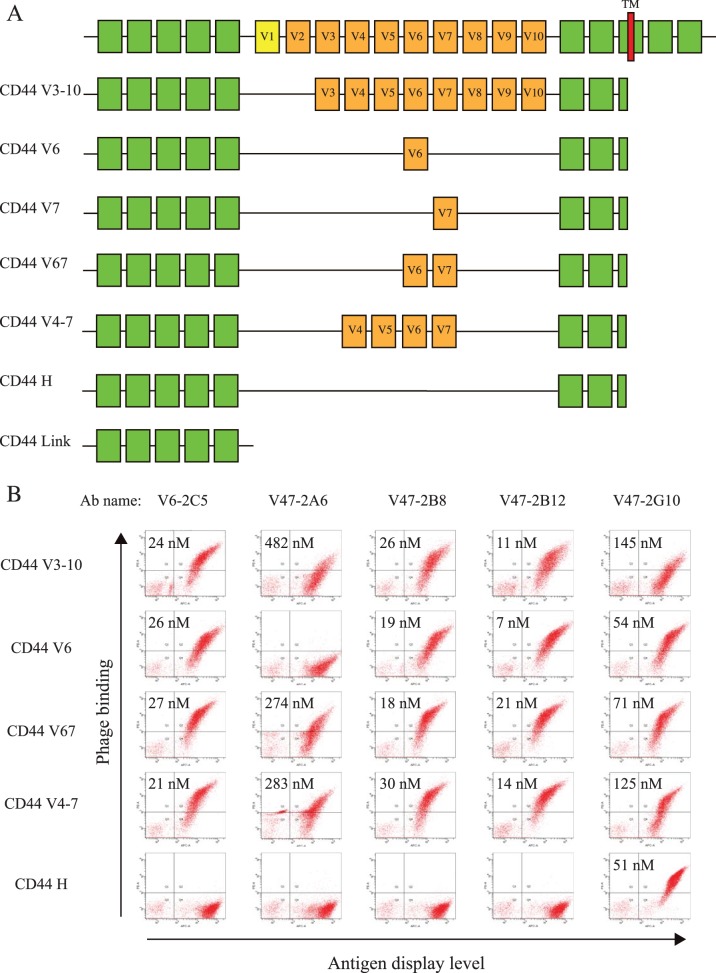
Characterization of five CD44 antibodies using a panel of CD44 variants displayed on the yeast cell surface. A) Diagram of the yeast displayed CD44 variant domains; B) binding specificity and affinity of anti-CD44 scFv assessed on the yeast displayed domains by flow cytometry.

Of the unique mAbs generated, we examined those binding CD44 in detail due to the importance of this antigen in tumor biology. Expression of multiple isoforms of CD44 has been identified in tumors [31]–[38]. To distinguish mAbs that are selective for different CD44 isoforms, we displayed 5 splice variants (CD44 V6, CD44 V7, CD44 V67, CD44 V4-7, CD44 V3-10), and 2 standard forms (CD44 link domain and CD44 H) on the yeast surface (Figure 4a). These yeast displayed CD44 forms were used for Ab library selection, mAb screening, as well as evaluation of Ab binding specificities and affinities. MAbs binding specifically to the V6, V7, or the constant domains with affinities ranging from 7 to 482 nM were identified from the phagemid library (Figure 4b). The CD44 domain binding profile suggested that antibody V6-2C5, V47-2B8, V47-2B12 bound CD44 v6, V47-2A6 bound CD44 v7, and V47-2G10 bound the CD44 standard form at the membrane proximal region.

**Figure 5:**
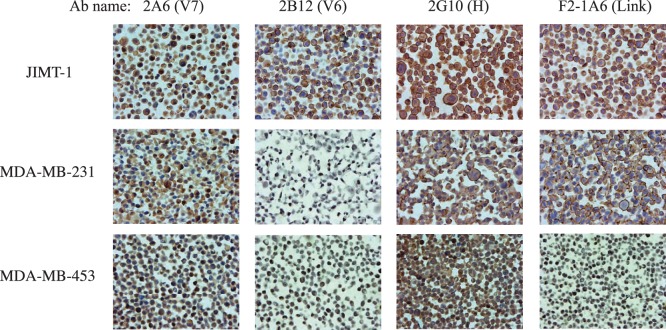
Immunohistochemistry staining of breast cancer cell buttons using CD44 mAbs. Breast cancer cell lines with known CD44 expression levels were processed to make paraffin embedded cell buttons, and used to evaluate anti-CD44 mAb staining patterns. Abs studied included V7 specific mAb 2A6, V6 specific mAb 2B12, CD44 standard form binding mAb 2G10, and CD44 link domain specific mAb F2-1A6.

**Figure 6:**
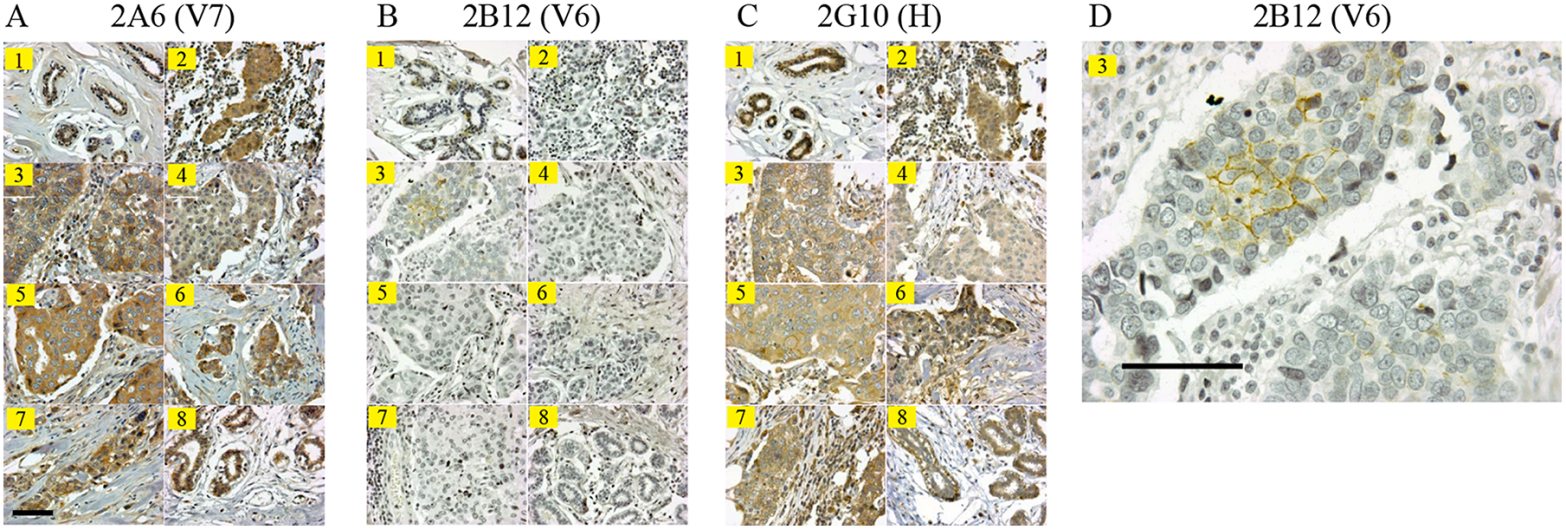
Breast tumor tissue array staining by immunohistochemistry with CD44 mAbs. A tissue array containing samples from 8 different patients (numbered 1–8) was evaluated after staining with different CD44 mAbs. A) V7 specific mAb 2A6, B) V6 specific mAb 2B12, C) CD44 standard from binding mAb 2G10, D) an enlarged view of tissue #3 stained with 2B12 showing distinct membrane bound CD44v6 in the center of a subset of cancerous cells. Magnification is 400x, and the scale bar is 50 µm.

The anti-CD44 phage antibodies with the highest affinities were converted to full length IgG which were used to stain breast cancer cell lines (Figure 5), and to detect specific variant in the breast tumor sections (Figure 6). Both MDA-MB-231 and JIMT-1 [39] are known to express CD44 at high level, and MDA-MB-453 cells express lower levels of CD44 [40]. The V6 specific mAb 2B12 showed membrane binding on JIMT-1 cells but not MDA-MB-231 or MDA-MB-453 cells. The link domain specific mAb F2-1A6 showed positive binding on both JIMT-1 and MDA-MB-231, but not MDA-MB-453 cells, consistent with prior flow cytometry analysis [23]. The CD44-H specific mAb showed the strongest signal, while the V7 specific mAb showed a weaker signal, and both antibodies showed some intracellular signal in MDA-MB-453 cells.

Breast tumor sections stained with three antibodies, 2A6, 2B12, and 2G10, also demonstrated unique CD44 staining patterns. The V7 and H specific Abs showed diffusing staining with epithelial cells including the normal mammary duct, the V6 specific 2B12 antibody stained a subset of the tumor epithelial cells with a distinctive membrane pattern (Figure 6). The biological relevance of V6 within the tumor tissue is unknown.

**Figure 7:**
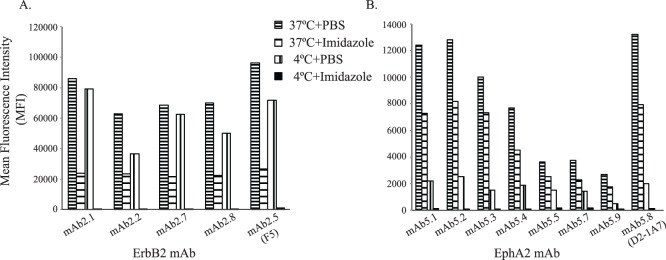
Internalization of mAbs into cancer cells as determined by chelated ligand-mediated internalization assay (CLIA). Ni-NTA liposomes loaded with dye were internalized into cells mediated by hexa-histidine tagged scFv mAbs prebound to the cell surface at 37°C compared to 4°C. Imidazole buffer was used to remove the surface bound dye-loading liposomes by disrupting the Ni-NTA and hexa-Histidine interaction. A) Internalization of ErbB2 mAbs into ErbB2 expressing SKBR3 cells; B) internalization of EphA2 mAbs into EphA2 expressing MDA-MB-231 cells.

Other cell binding antibodies were also validated for cell staining and internalization. Ab internalization was evaluated using the chelated ligand-mediated internalization assay (CLIA). Specifically, the histidine-tagged scFv coupled with the Ni-NTA coated fluorescent liposome bound the target receptor on the intact mammalian cell surface at 4°C compared to that internalized into the cells at 37°C. The surface bound liposomes were removed by imidazole wash showing no fluorescence signal left for the 4°C incubation group, while the internalized liposomes remained for the 37°C incubation group (Figure 7). The ratio of fluorescence between the imidazole and the PBS wash reflected the fraction of internalized Abs. Both monoclonal scFv antibodies to ErbB2 domain 1 and EphA2 ECD were internalized into SKOV3 and MDA-MB-231 cells, respectively (Figure 7). Some mAbs showed comparable internalization to mAbs F5 and D2-1A7, which bound ErbB2 and EphA2, respectively, but were isolated from selection for internalization into intact cells [23], [41].

**Figure 8:**
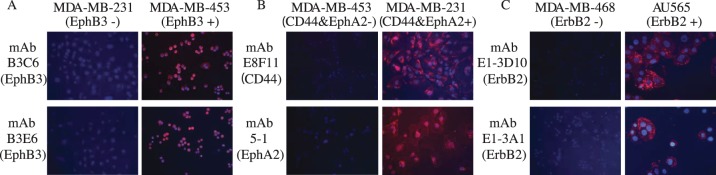
Binding of mAbs to cancer cell lines that overexpress the target antigens as determined by fluorescent microscopy. A) EphB3 mAbs showed positive staining on EphB3 overexpressing cell MDA-MB-453, B) CD44 and EphA2 mAbs showed positive staining on MDA-MB-231 cells, C) ErbB2 domain 1 binding mAbs showed positive staining on the ErbB2 overexpressing cell AU565.

Breast cancer cell lines known to express the target antigens ErbB2, EphA2, EphB3, and CD44 with high and low levels were stained with the identified phage mAbs and evaluated by fluorescence microscopy. Consistent with the gene expression data [40], EphB3 antibodies stained MDA-MB-453 cells, CD44 and EphA2 antibodies were positive on MDA-MB-231, and the ErbB2 domain 1 binders showed intensive intracellular staining of ErbB2 positive AU565 cells (Figure 8).

## Discussion

We have demonstrated that antigen yeast display combined with Ab phage display is a rapid method to generate highly specific mAbs to secretome proteins that recognize native proteins. Previously, we showed that the extracellular domains of EphA2 and CD44 can be displayed on the yeast cell surface and used to identify mAbs from a phage antibody library pre-selected on breast cancer cells overexpressing EphA2 and CD44 [23]. Here, we demonstrate the generality of this approach using un-selected naïve phage and phagemid antibody libraries. The approach presented here could be amenable to automation allowing high throughput antibody selection.

Yeast antigen display proved to be a robust system to display protein domains for antibody selection and screening based on the range of proteins we tested. The advantages over other antigen production system include 1) easy and fast cloning of antigen using gap repair, 2) simultaneous display of multiple domains, 3) high density of homogeneous antigens on cell surface, and 4) relatively clean background for antibody selection. Potential limitations of this method are the non-natural epitope space caused by mannosylation [42], [43] and inability to express multi-pass transmembrane proteins and GPI-linked membrane proteins.

The expression level of antigen domains was weakly correlated with the antigen size and more strongly correlated with the protein fold and the structure. Generally, the Ig fold and the Fn3 fold were better displayed in the yeast expression system, while the sema domain was difficult to display. Expression could potentially be improved with codon and domain optimization, changing the orientation of Aga2, or by including yeast chaperones [44].

The number of phage antibodies generated was directly correlated with the display level of the antigen, but not associated with the antigen size. A higher number of unique mAbs seemed to be generated from the phagemid library compared to the phage Ab library, suggesting that some Ab sequences were lost during the construction of the smaller phage library by sub-cloning from the phagemid library [29], [30].

A number of the mAbs we generated were able to recognize the native antigen as presented on the cell surface. Interestingly, we identified identical mAbs that we previously identified using direct selection on intact cells, namely F5 and D2-1A7, which binds HER2 and EphA2, respectively [23], [41]. This observation confirms the native structure of the antigen as it is presented on yeast and suggests similar selection pressures.

The yeast display of antigen domains allowed flexible, rapid, and easy expression of multiple antigen domains as well as direct affinity assessment by flow cytometry [28], which proved extremely useful for CD44 splice variants where the soluble proteins are not available. We displayed seven different variant forms of CD44 on the yeast surface, and completed the phage antibody library selection and screening within a month. The resulting mAbs have affinities ranging from 7 nM to 482 nM, were differentially specific to different domains, and could stain breast tumor sections.

MAb internalization via receptor-mediated endocytosis was evaluated for HER2 and EphA2 mAbs; the majority of mAbs to the HER2 domain 1 Abs and EphA2 Abs demonstrated intracellular staining as determined by CLIA [45] and fluorescent microscopy. The results indicate that the functionality of mAbs are associated with the methods of Ab selection, and can also be approached by using the correct epitope domain of the antigens, such as the HER2 domain 1.

The described approach may be challenging in the absence of knowledge of a target’s domain structure. However, the yeast system allowed us to rapidly display multiple domains with different starting and ending amino acids to search for the optimal domains. We also noticed that some antigen epitopes are difficult to recapitulate on the yeast cells as exemplified by the failure of mAbs isolated by using recombinant antigens to bind the yeast displayed domains. Such epitopes will definitely be missed using this approach.

We conclude that the yeast display of antigen domains and domain combinations are useful for isolating recombinant antibodies from naïve antibody libraries in a short time period starting from the antigen sequences. This platform will allow Ab discovery for a large function of the antigen epitope space, but not all the epitopes, which will need other approaches to complete Ab discovery.

## Materials and Methods

### Cell lines, media, antibodies and full-length cDNA clones

Breast cancer cell lines A431, AU565, Colo205, JIMT-1, MDA-MB-231, MDA-MB-453, MDA-MB-468, SKBR3, SKOV3, SUM159PT and HEK293A cells were obtained from the American Type Culture Collection (ATCC). The cell lines were cultured using conditions described previously [46]. The yeast strain EBY100 was grown in YPD medium (*Current Protocols in Molecular Biology, John Wiley and Sons, Chapter 13.1.2*). EBY100 was transfected with expression vector pYD2 [47] and was selected on SD-CAA medium [48] (*Current Protocols, Chapter 13*). The Aga2p antigen fusion was expressed on the yeast surface by induction in SG-CAA medium (identical to SD-CAA medium except the glucose is replaced by galactose) at 18°C for 24–48 hr as described previously [49]. *E. coli* strains DH5α and TG1 were used for the preparation of plasmid DNA and the expression of soluble scFv antibodies respectively. SV5 antibody was purified from hybridoma supernatant using Protein G and directly labeled with Alexa-488 or Alexa-647 using a kit provided by the manufacturer (Invitrogen; Carlsbad, CA). Biotin conjugated rabbit anti-fd bacteriophage (Sigma), biotin conjugated anti-rabbit (Vector Labs), Streptavidin Phycoerythrin (PE) (Biosource/Invitrogen), streptavidin Texas Red (GE Healthcare), streptavidin HRP conjugate were used to detect antibodies. The full-length cDNAs were obtained from the ATCC and Open Biosystems.

### Cloning of protein domain coding genes into the yeast display vector

For antigen display, the cDNA encoding the full length antigen or antigen domain was amplified by PCR using primers that anneal to the 5′ and 3′ ends of the antigen cDNA and with 25 nucleotide overlaps with the yeast display vector pYD2 digested with the restriction enzymes NcoI and NotI. The antigen DNA fragment was then cloned into NcoI-NotI-digested pYD2 vector by using gap repair [50], [51]. Gap repair allows cloning of virtually all cDNA fragments including those with internal digestion sites.

Primers for cloning the target antigen gene by gap repair:

- Ag-Gap5 primer 5′-GTTGTTCTGCTAGCGGGG**CCATGG**---3′
- Ag-Gap3 primer 5′-TTCGAAGGGCCCGCCT**GCGGCCGC**---3′

The 5′ sequence indicated anneals to Nco1-Not1 digested pYD2 DNA for cloning by gap repair, the bolded sequences are the Nco1 and Not1 sites. The dashed sequence should include approximately 24 nucleotides that anneal to the 5′ and 3′ ends of the target antigen DNA.

### Display of protein domains on the surface of yeast cells

Multiple target genes were amplified by PCR in 96-well plates using high-fidelity DNA polymerase, and used to transform LiAc treated EBY100 cells together with NcoI/NotI digested vector pYD2 using the TRAFO method with gap repair in 1 ml 96-well plate [50], [51]. Specifically, TRAFO mix including the vector with large enough quantity for 96 transformation was prepared and used to resuspend the EBY100 yeast cells followed by aliquoting 359 µl of TRAFO-treated cells into each well that contains 1 µl of the target gene PCR fragment. After incubation for 30 min at 30°C followed by 45 min at 42°C, the cells were pelleted by centrifugation at 3000 rpm for 2 min, the transformation mix removed, cells washed once in 1 ml of sterile water, 1/10 of the cells transferred to a new 1 ml deep 96-well plate containing 600 µl of SD-CAA media, and cultured for 24 hrs at 30°C. Dilution of untransformed yeast cells was repeated by growing 1/10 of 24 hr culture in a new 1 ml 96-well plate containing 600 µl of SD-CAA media followed by induction culture in SG-CAA media for 24 hrs at 18°C.

### Quantitation of protein density on yeast surface by flow cytometry

A fraction of the induced yeast cells were transferred into a V-bottom 96-well plate, washed once by resuspending in 100 µl of FACS buffer, followed by centrifugation, aspiration of the supernatant, resuspension in 50 µl of FACS buffer, and stained by incubation with 50 µl of 1 µg/mL Alexa-647 labeled anti-SV5 IgG diluted in FACS buffer for 1 hr at 4°C. After washing the stained yeast cells once in 200 µl of FACS buffer, the cell fluorescence was measured in a FACS LSRII flow cytometer using Quantum™ Alexa Fluor 647 bead (Bangs Laboratories Inc.) as reference to calculate the protein density on each cell.

### Selection of phage antibody library against yeast displayed protein domains

A phage display naïve human single chain library was used for target specific antibody selections [29], [30]. The induced yeast cells displaying an human Fc domain were used to deplete the non-specific binders by incubating 1×10^13^ phage particles with 10^9^ yeast cells for 2 hr at 4°C. The filtered supernatant containing the depleted phage library was then aliquoted into each well of 1 ml 96-well plate containing about 1×10^7^ yeast cells displaying individual target protein domains for 1 h at 4°C. Yeast cells were washed ten times with cold PBS and pelleted by centrifugation. The bound phage antibodies were eluted by incubating yeast cells with 100 µl of 100 mM triethylamine, neutralized with 50 µl of 1 M Tris-HCl (pH 7.4), and used to infect exponentially growing *E. coli* TG1 as described previously [52]. Specifically, half of the phage eluent was transferred to a new 1 ml 96-well plate, incubated with 1 ml of *E. coli* TG1 cells for 30 min at 37°C, and 1/10 of the infected TG1 transferred to a new 1 ml 96-well plate containing 2YT/Amp/2% glucose, and cultured for 16 hrs at 30°C. A small fraction of the overnight culture was transferred to another 1 ml 96-well plate containing 150 µl of 2YT/Amp/0.1% glucose using 96-pin transfer device followed by culture for 2.5 hr at 37°C, infection with VCSM13 for 30 min at 37°C, and culture in additional of 450 µl of 2YT/Amp for 16 hrs at 30°C. In the case of fd phage library, the TG1 infected phage eluent was cultured in 2YT/Tet for 16 hrs at 30°C.

The resulting phage output of each target was harvested, precipitated using 20% PEG6000/1.5 M NaCl, resuspended in 100 µl of cold PBS, and subjected to another round of selection to the corresponding target protein domain.

For the second round of selection, the 100 µl of output phage from the round 1 selection was depleted in 1 ml 96-well plate by incubating with 10^8^ yeast cells displaying an human Fc domain for 2 hr at 4°C followed by centrifugation to pellet the yeast cells. The supernatant of each well was transferred to a new 1 ml 96-well plate, and mixed with 1×10^7^ of yeast cells displaying the corresponding target protein domains for 1 h at 4°C. The rest of the selection procedure was the same as the first round. If needed, the third round of selection can be performed following the same procedure as the second round of selection. When the selection was completed, each selection output can be plated for monoclonal phage analysis.

### Evaluation of phage antibody binding to yeast-displayed protein domains

Polyclonal phage antibodies from each round of selections were evaluated by flow cytometry for binding to the corresponding target protein domains displayed on yeast cells. In detail, the supernatant of phage culture in 96-well plate was transferred to V-bottom 96-well plate and incubated with yeast cells displaying the corresponding target antigen for 2 hrs at 4°C. The bound phage antibodies were detected by incubating cells with biotin conjugated anti-fd antibody (1 µg/ml) for 30 min at 4°C and streptavidin-PE followed by flow cytometry analysis. The binding of phage antibodies to the yeast displayed target antigens were represented by the percentile of yeast population in the Q2 quadrant of the dot plot.

### Characterization of phage antibodies

After two or three rounds of selection, 96 individual colonies from each target specific selection were isolated and the phage prepared by growing the single colonies in 96-well microtiter plates as described [53]. Binding of each phage antibody to yeast displayed antigen was determined by incubation of 10^5^ yeast cells with 100 µl phage supernatant diluted in FACS buffer (PBS with 1 mM MgCl_2_, 0.1 mM CaCl_2_ and 0.3% BSA) for 2 h at 4°C in conical 96-well microtiter plates, followed by incubation with biotinylated anti-fd antibody and streptavidin-phycoerythrin conjugate, and analyzed using a FACS LSRII (Becton Dickinson). The number of unique phage antibodies was determined by 96-well RCA reaction followed by DNA sequencing.

### Expression of soluble antibodies as scFv and IgG

The scFv coding genes from the identified phage antibodies were subcloned from the phage-display pHEN1 [52] via NcoI-NotI into expression vector pSyn1 for scFv expression [54], or AbVec with rabbit Fc for IgG expression [55]. scFvs were produced in the periplasm of *E. coli* strain TG1, and purified by osmotic shock and immobilized metal affinity chromatography, as reported previously [54]. IgG proteins were secreted in the media by transfected HEK293A cells as described elsewhere [55], and purified by Protein A affinity chromatography (GE Healthcare). The homogeneity and purity of the protein preparations were verified by SDS-PAGE stained with coomassie blue; protein concentrations were measured by micro-bicinchoninic acid assay (Pierce, Rockford, IL).

### Binding affinity assay by flow cytometry

Each scFv was incubated with 1×10^5^ yeast cells induced to express the target protein domain for 1 hr-16 hrs at the indicated concentration, or the target antigen overexpressing cancer cells [28], [56]. Cell binding was performed at 4°C in PBS containing 0.3% BSA in volume sufficient to maintain constant antibody concentration for equilibrium conditions. After two washes with 200 µl of PBS, bound scFv was detected by the addition of 100 µl (1 µg/ml) of biotinylated His probe (Santa Cruz Biotech.) and streptavidin-PE (Biosource/Invitrogen), and the yeast displayed Ag co-stained with Alexa 647 labeled anti-SV5. After incubating 30 minutes at 4°C, the cells were washed twice and resuspended in PBS containing 4% paraformaldehyde. Fluorescence was measured by flow cytometry in a FACS LSRII (Becton Dickinson), and median fluorescence intensity (MFI) was calculated using Cellquest software (Becton Dickinson). Equilibrium constants were determined as described [57], except that values were fitted to the equation MFI = MFI_min_ + MFI_max_* [Ab]/(K_D_+[Ab]) using Kaleidagraph (Synergy Software).

### Antibody internalization assay

The anti-ErbB2 and anti-EphA2 scFv antibodies were evaluated for the ability to internalize into human breast cancer cells SKBR3 and MDA-MB-231 cells, respectively, by chelated ligand-mediated internalization assay (CLIA) as described previously [45]. Briefly, the cells were grown to 80–90% confluence in the media recommended by ATCC supplemented with 10% fetal calf serum (FCS) and harvested by trypsinization, seeded at 10,000/well in 96-well plates, and incubated overnight at 37°C. The next day, Ni-NTA liposomes (0–1 mM total phospholipid) were incubated for 4 hrs with the cells along with the (His) 6-containing scFv (20 µg/mL unless otherwise indicated) in 100 µL tissue culture media supplemented with 10% FCS under four different conditions. The conditions are 37°C followed by PBS wash, 37°C with 250 mM phosphate buffered imidazole wash, 4°C with PBS wash, and 4°C with 250 mM phosphate buffered imidazole wash. Cells were then trypsinized, washed, and the fluorescence measured using a FACS LSRII (Becton Dickinson).

### Immunohistochemistry

Three breast cancer cell lines, MDA-MB-231, MDA-MB-453, and JIMT-1, were used to make paraffin embedded cell button at UCSF tissue core, and stained with CD44 antibodies following standard IHC protocol.

De-identified human breast tumor tissue samples were obtained from University of California, San Francisco, Department of Pathology archives. Samples were collected with consent under UCSF IRB# 11-06720 “Retrospective Study of Adjunctive Pathologic Diagnostic Features, Biomarkers Expression, and Molecular Alterations in Early Breast Neoplasms and in Morphologic Mimics”. The UCSF Mount Zion Panel Institutional Review Board approved the use of this archived patient tissue samples in research and waived the need of consent.

Breast tumor tissue samples were arrayed, and cut to five-micrometer sections. After antigen retrieval, the samples were stained with three different anti-CD44 IgG antibodies with rabbit Fc, 2A6, 2B12, and 2G10, respectively, followed by staining with biotin-conjugated anti-rabbit secondary antibody (Vector Labs Co.), ABC complex (Vector Labs Co.), and standard DAB color development (Vector Labs Co.) sequentially. A pathologist (YYC) evaluated the staining result.

### Immunofluorescence

MDA-MB-231, MDA-MB-453, MDA-MB-468, and AU565 cells were grown on coverslips to 70% of confluence in 12 well-plates and incubated with 10^11^ phage antibodies for three hours at 37°C. The coverslips were washed once with PBS, twice with PEM (80 mM Potassium PIPES (pH 6.8), 5 mM EGTA (pH 7), 2 mM MgCl_2_), and fixed with PEM containing 4% (W/V) paraformaldehyde for 30 min on ice. Cells were quenched with 0.1 M NH_4_Cl, permeabilized with 0.5% Triton X-100, and blocked with 5% non-fat dry milk in TBS-T buffer overnight at 4°C. After blocking endogenous biotin with Avidin-Biotin Kit (Lab Vision), intracellular phages were detected with biotinylated anti-fd polyclonal antibody (Sigma) and streptavidin Texas Red (GE healthcare). Coverslips were inverted on a slide on mounting medium and microscopic images were taken with a Zeiss fluorescence microscope (Zeiss, Germany).
